# Mental health outcomes in parents of children with a cancer diagnosis in Sweden: A nationwide cohort study

**DOI:** 10.1016/j.eclinm.2022.101734

**Published:** 2022-11-17

**Authors:** Yishan Liu, Jan Sundquist, Kristina Sundquist, Deqiang Zheng, Jianguang Ji

**Affiliations:** a Center for Primary Health Care Research, Department of Clinical Sciences Malmö, Lund University, Sweden; b Department of Family Medicine and Community Health, Department of Population Health Science and Policy, Icahn School of Medicine at Mount Sinai, New York, USA; c Center for Community-based Healthcare Research and Education (CoHRE), Department of Functional Pathology, School of Medicine, Shimane University, Japan; d Department of Epidemiology and Health Statistics, School of Public Health, Capital Medical University, Beijing, China

**Keywords:** Childhood cancer, Parents, Mental health, ITS, CI, confidence interval, RR, rate ratio, CCI, Charlson Comorbidity Index, ITS, interrupted time series

## Abstract

**Background:**

The diagnosis of paediatric cancer is a crisis for the parents who are the primary caregivers of the affected child. A comprehensive assessment of the longitudinal impact of childhood cancer on parental mental health and the potential sex differences between the parents is lacking. Thus, we aimed to explore the subsequent short- and long-term mental health outcomes among the parents of children with cancer and examine whether the outcomes vary between the mother and father.

**Methods:**

By combining several Swedish registers, parents of a child (ages 0–14 years) with a cancer diagnosis between Jan 1, 2006, and Dec 31, 2016 were identified. For each parent of children with cancer, up to five mothers or fathers of cancer-free children were randomly selected and matched, respectively. Hospital contacts for any mental health disorders between 5 years before and 7 years after the diagnosis of childhood cancer were retrieved. An interrupted time series negative binomial regression was performed to assess the short- and long-term impact of a childhood cancer diagnosis on the parents’ subsequent mental health outcomes.

**Findings:**

16,199 mothers (2852 with a child with cancer and 13,347 without) and 15,708 fathers (2769 with a child with cancer and 12,939 without) were included in this study. Compared with mothers of children without cancer, mothers of children with cancer had higher risks of mental health disorders in the first year after diagnosis (rate ratio [RR] and 95% Confidence Interval [CI], 1.17 (1.03–1.32)), and notably, the adverse impact became more severe over time (RR and 95% CI, 1.36 (1.07–1.74), in the seventh year). For fathers of children with cancer, the risk of mental health disorders was continuously higher compared to matched comparisons (RR and 95% CI, 1.31 (1.01-1.71)).

**Interpretation:**

Our findings suggested that parental mental health was affected continuously by a diagnosis of childhood cancer in their children. In particular, the mother's mental health was affected more severely. Customised psychological services or interventions are highly needed for the parents of children with cancer.

**Funding:**

10.13039/501100004359Swedish Research Council, Allmänna Sjukhusets i Malmö Stiftelsen för bekämpande av cancer, 10.13039/501100003793Swedish Heart-Lung Foundation, ALF funding from Region Skåne and 10.13039/501100004543China Scholarship Council.

**Research in context**

**Evidence before this study**

The previous studies published in English were searched via PubMed between Jan 1, 2000, and June 30, 2022, using the title terms “childhood”, “pediatric”, “cancer”, “neoplasm”, “parents”, “caregiver”, “mental”, “psychiatric”. Most previous studies focused on the impact of childhood cancer on patients themselves or the parental short-term health outcomes. But the knowledge is limited on the time trend of longitudinal impact and the potential sex differences between mothers and fathers of children with cancer.

**Added value of this study**

Using the Swedish nationwide high-quality longitudinal registers data, we conducted a thorough analysis to explore the patterns of mental health outcomes in parents of children with cancer. Our study provides the novel finding that different trajectories of long-term mental health outcomes were observed in mothers and fathers. The adverse effect on fathers was relatively stable and constant, whereas the adverse effect became continuously worse for mothers. Additionally, our study identified the most vulnerable subgroups in parents of children with cancer and specific mental disorders that were affected.

**Implications of all the available evidence**

To support parents in better overcoming the challenge of children being diagnosed with cancer, and to safeguard their mental well-being, especially among high-risk groups of parents, customised psychological services or interventions are greatly needed.

## Introduction

Childhood cancer is the most common cause of death by disease among children in Sweden^1^ and many other high-income countries. The adverse impacts of childhood cancer on survivors have been quite extensively explored in previous studies.^2^, ^3^, ^4^, ^5^ However, childhood cancer is not only traumatic for the patients themselves, but also for their parents.^6^

Parents, central in paediatric care, are often involved in making decisions on multiple treatment options, closely monitoring the child's symptoms, and reconciling frequent and extended hospital visits.^7^ Long-term care is required to overcome the potential physical impairment and the increased risk of late effects for children.^8^^,^^9^ It's particularly challenging for parents to face the fear of the potential death of their child, overcome the conflict between work and childcare, and deal with the emotional and practical demands that arise from medical and non-medical care and expenditures.^10^^,^^11^ The high levels of burden for the parents who care for a cancer-affected child can compromise their mental health.^12^ Previous studies found that compared to the general population, parents of children with cancer often experienced post-traumatic stress,^13^ uncertainty, anxiety, and depression over the first year of treatment.^14^^,^^15^ The worse parental mental health conditions might last even years after treatment.^15^, ^16^, ^17^, ^18^, ^19^ Among the available evidence, the mental health symptoms were usually self-reported, the sample sizes were relatively small, and the long-term effects were paid less attention to. Two previous studies explored the association of the diagnosis of childhood cancer with the rates of parental hospital contacts for clinically diagnosed mental health disorders.^18^^,^^19^ Both studies showed a high risk of hospital contact for psychiatric disorders among parents of cancer-affected children during a long follow-up time. The study conducted in Canada did not explore the risk in the fathers of children with cancer, and only outpatient visits were available in this study.^19^ In the study conducted in Denmark, they did not explore the potential longitudinal variations of mental health disorders after the diagnosis of childhood cancer.^18^^,^^20^ Both studies did not assess the potential difference between the case and control parents regarding their mental health disorders before baseline. As the underlying difference may affect parental subsequent mental health outcomes, the study results may be biased.

With the timely diagnosis and use of advanced treatments, the relative-high survival rates of childhood cancer in Sweden^21^ led to a growing number of parents of cancer-affected children. However, a survey conducted by the Swedish Childhood Cancer Foundation suggested that more than 50% of the parents of childhood cancer survivors experienced insufficient psychological support.^22^

Thus, this study aimed to (A) explore the short- and long-term mental health outcomes among parents of children with cancer, and (B) examine the potential heterogeneity between the mothers' and fathers’ responses to the challenges of caring for a cancer-affected child.

## Methods

### Study population

Children aged 0–14 years with primary cancer diagnosed between Jan 1, 2006, and Dec 31, 2016, were identified from the Swedish Cancer Register. We further identified the parents of these children through the Multi-Generation Register and excluded records where information on neither the mother nor the father was available. If more than one child in a family was diagnosed with cancer, we kept the record of the older child. Two separate data sets were created, one with information on the mothers of all children with cancer, and the other with information on the fathers. For each case parent with an affected child, up to five corresponding parents whose children were not diagnosed with cancer were randomly selected and matched. The matching procedure is conditional on the child's age at the year of diagnosis (age difference should be less than six months), the child's sex (should be the same), the age of the parents (less than one year apart), the country of origin (Sweden or abroad) and the municipality (should be the same).

### Assessment of exposures and outcomes

The date of birth of parents and children, country of origin, and the municipality of living on the index date were retrieved from the Swedish Multi-Generation Register and the Total Population Register. Country of origin was classified into two groups: Sweden and other countries. Based on geography and population, we divided the municipality of living into three places of living: big cities, other northern cities, and other southern cities.

Apart from the variables used for matching, another parental demographic characteristic, i.e., education level (<10, 10–12, or >12 years of education), that might affect the outcomes, was adjusted in the final regression model. This variable was extracted from the Longitudinal Integration Database (LISA). In addition, the Charlson Comorbidity Index (CCI) score, as a continuous variable, was calculated at baseline for parents by identifying specific comorbid conditions in the National Patient Registry^23^ from the first record until the index study day. The index dates for both cases and control parents were defined as the diagnosis date of the child's cancer (case).

Hospital contacts for mental health disorders were retrieved from the National Patient Registry. The registry includes data on nationwide inpatient hospitalisations for mental health disorders, completed since 1987, and nationwide outpatient visits including psychiatric care from both private and public caregivers, completed since 2001. The study observation ended on December 31, 2018, when the most recent and complete data was available by us. The reporting system works well and delivers information to the register once a month. The submitted data is always tested and corrected if the data are suspected to contain erroneous or invalid data points.^24^ ICD-10 codes from F00 to F99 were used to define mental health disorders.

In this study, the main outcome was the number of occurrences of hospital contact with the main diagnosis of any mental health disorder for each parent during the observation period. To adjust for the potential difference between the parents before the diagnosis of childhood cancer, we identified all the mental health disorder hospital contacts that were recorded five years before the index study date. All the parents were followed until the seventh year after the index date, at the date of death (linked to the Swedish Death Register), or at the end of the study period (Dec 31, 2018), whichever came first.

### Stratification of exposures and outcomes

Given the differences in care between inpatient admission and outpatient clinical diagnoses, we divided the total hospital contacts into two types, inpatient hospitalisation, and outpatient visits. The most common cancer type of childhood cancer was leukaemia, followed by nervous system tumours and non-Hodgkin lymphoma.^25^ To identify potential heterogeneity of cancer site and age at diagnosis of childhood cancer concerning parental risks of mental health disorders, we reassembled the childhood cancer sites into three subgroups: haematological malignancies; tumours in the nervous system; and other malignancies, as well as the child's age at diagnosis into the subgroups 0–4, 5–9 and 10–14 years.

Furthermore, to provide insight into the association between the event (child cancer diagnosis) and specific mental health outcomes, subgroup analyses were performed for several subtypes of mental health disorders in the parents, including alcohol abuse (ICD-10 code as F10), drug abuse (ICD-10 code as F19), severe depressive disorder (ICD-10 code as F32.2 or F32.3), recurrent depressive disorder (ICD-10 code as F33), and adjustment disorder (ICD-10 code as F43).

### Statistical analysis

The comparability of baseline demographic characteristics was assessed by calculating a standardised difference between the parents of children with cancer and the matched comparisons. The standardised difference with a value of less than 10% indicates the similarity of variables between the two groups.^26^

Based on the data in the National Patient Register, the number of hospital contacts with mental health disorders as the main diagnosis was counted every year per parent. Observations began five years prior to the index date and continued up to seven years after the cancer diagnosis in children. The secular trends in hospital contact rates were assessed separately for mothers and fathers with and without children with cancer. The annual rate of mental health disorders was calculated as the number of hospital contacts divided by the actual person-years per follow-up year.

We then used an interrupted time series (ITS) analytic framework to analyse pre- and post-changes in hospital contacts due to mental health disorders upon cancer occurrence in the child in the mothers and fathers, separately. The ITS model is an extension of repeatedly measured generalised estimating models. It incorporates interactions among groups, events, and time, providing a way to test the effect of an ‘interruption’ that occurs at a known point in time during the observation period.^27^^,^^28^ It deals well with multiple outcome measures and longitudinally tracks the outcomes both before and after the child had been diagnosed with cancer.^29^ To better fit the counted data of the hospital contacts, the equation was revised to regress the outcome of a negative binomial distribution with a logarithm link function within the ITS framework.

$$\text{Log}\left(\theta \right)=\beta_{0}+\beta_{1}\text{∗group}+\beta_{2}\;\text{∗}\;\text{time}+\beta_{3}\;\text{∗}\;\text{group}\;\text{∗}\;\text{event}+\beta_{4}\;\text{∗}\;\text{time}\;\text{∗}\;\text{group}\;\text{∗}\;\text{event}+X^{T}\text{η }$$

The general equation of the model in our study is written above, where θ is the mean of the negative binomial distribution; $\beta_{1}$ indicates the baseline difference between two groups; $\beta_{2}$ indicates the population-based time-varying effect of mental health disorder diagnoses; $\beta_{3}$ indicates the effect of a short-term shock to case parents; $\beta_{4}$ indicates the effect of a long-term shock to case parents; X is the vector of covariates and η are the effects of corresponding covariates. We were mainly interested in the statistical measure of parameters $\beta_{3}$ and $\beta_{4}$. From the perspective of our study, we could interpret these two parameters as two questions: (1) whether it had any abrupt change in levels of parental hospital contact for mental health disorders in the year of childhood cancer diagnosis; and (2) whether the event of a child being diagnosed with cancer would result in a gradual slope change in parental mental health disorder hospital contacts in a comparison with the matched parents.

Estimates and 95% confidence intervals (CIs) for the rate ratio (RR) in parents of children with cancer, compared to the matched comparisons, were calculated to assess the short- and long-term impact on their mental health.

Furthermore, factors potentially associated with the outcomes, i.e., the country of origin, parental living place, education level, parents' age, child's sex, and CCI scores, were included in multivariate analyses. In addition, subgroup analyses for childhood cancer sites, the child's age at diagnosis, and the mental health disorders of interest were conducted.

For all analyses, a two-tailed *P* value < 0.05 was considered statistically significant. All statistical analyses were performed using SAS version 9.4 (SAS Institute, Cary, NC, USA).

### Patient and other consent

This nationwide retrospective cohort study was approved by the Ethical Review Board in Lund (number 2012/795 and later amendments). All the individuals involved in this study had pseudonymised serial numbers in the database to protect their integrity. Patient consent was not required for this study. The project database is located at the Center for Primary Health Care Research at Lund University in Malmö, Sweden.

### Role of the funding source

The funders had no role in the study design, data collection, data analysis, finding interpretation, or the writing of the manuscript. All authors had access to the data in the study and took the decision to submit the study results for publication.

## Results

In total, we identified 2852 mothers and 2769 fathers whose children were diagnosed with cancer. We further randomly selected 13,347 mothers and 12,939 fathers of cancer-free children as the reference groups and matched by the child's sex, age, parental age, and country of origin, as well as the place of living when the child was diagnosed with cancer (Table 1). Among the parents, the mean age for mothers was 37.0 years for both cases and controls, whereas the mean age for fathers was 39.8 years for cases and 39.6 years for controls. The median (IQR) follow-up time was seven years (4.7-7.0) for both parents of children with and without cancers.

**Table 1:** Basic demographic and clinical characteristics among parents of children with cancer and the matched comparisons.

	Parents of children with cancer, n (%)	Matched comparisons, n (%)	Standardised mean difference (%)^a^
Mothers	2852	13,347	
Maternal age at the index date			
<30	416 (14.6)	1889 (14.2)	3.17
30-39	1526 (53.5)	7254 (54.3)	
≥ 40	910 (31.9)	4204 (31.5)	
Mean (SD)	37.0 (6.5)	37.0 (6.3)	0.24
Country of birth			
Sweden	2232 (78.3)	10,669 (79.9)	−4.12
Abroad	620 (21.7)	2678 (20.1)	
Place of living			
Big Cities	529 (18.5)	2643 (19.8)	3.62
Northern Sweden	387 (13.6)	1679 (12.6)	
Southern Sweden	1936 (67.9)	9025 (67.6)	
Education level (years)			
≤ 9	292 (10.2)	1251 (9.4)	3.54
10-12	1133 (39.7)	5405 (40.5)	
>12	1427 (50.0)	6691 (50.1)	
Baseline CCI score			
No comorbidities (0)	2555 (85.6)	12,094 (90.6)	4.02
Mild (1–2)	267 (9.4)	1160 (8.7)	
Moderate (3–4)	15 (0.5)	45 (0.3)	
Severe (≥5)	15 (0.5)	48 (0.4)	
Fathers	2769	12,939	
Paternal age at the index date			
<30	194 (7.0)	867 (6.7)	2.07
30-39	1278 (46.2)	6074 (46.9)	
≥ 40	1297 (46.8)	5998 (46.4)	
Mean (SD)	39.8 (7.0)	39.6 (6.7)	1.87
Country of birth			
Sweden	2197 (79.3)	10,444 (80.7)	−3.44
Abroad	572 (20.7)	2495 (19.3)	
Place of living			
Big Cities	521 (18.8)	2594 (20.0)	2.57
Northern Sweden	364 (13.2)	1633 (12.6)	
Southern Sweden	1884 (68.0)	8712 (67.3)	
Education level (years)			
≤ 9	330 (11.9)	1513 (11.7)	<0.01
10-12	1335 (48.2)	6255 (48.3)	
>12	1104 (39.9)	5171 (40.0)	
Baseline CCI score			
No comorbidities (0)	2480 (89.6)	11,827 (91.4)	5.75
Mild (1–2)	260 (9.4)	1009 (7.8)	
Moderate (3–4)	18 (0.7)	51 (0.4)	
Severe (≥5)	11 (0.4)	52 (0.4)	

a The standardised difference between the parent of children with cancer and the comparisons, with a value of 10% or less indicates appropriate matching.

### Secular trends in rates of mental health disorders

**Fig. 1:**
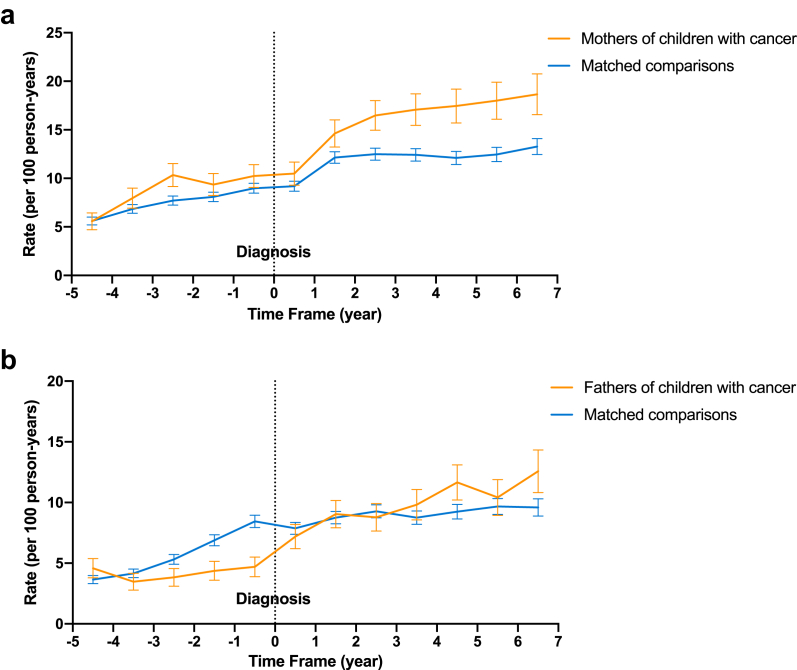
**Mental hospital contacts rate of parents before and after the diagnosis of childhood cancer (per 100 person-years).** (a), rate of mothers; (b), rate of fathers. Yellow lines represent the rates of hospital contacts for any mental health disorders per 100 person-years among parents of cancer-affected children from five years before to seven years after the index date. Blue lines represent the rates of hospital contacts for any mental health disorders per 100 person-years among parents of cancer-free children during the same observation period. The vertical dotted line represents the index year, i.e. the year the case child was diagnosed with cancer. Error bars indicate 95% confidence intervals (CIs) for each data point.

Generally, mothers and fathers in all groups had an increased rate of hospital contacts over time, as shown in Fig. 1. In the fifth year prior to diagnosis, the rates of mental health disorder hospital contacts were 4.59 cases per 100 person-years in fathers of children with cancer, 3.65 in the matched comparisons, 5.58 in mothers of children with cancer, and 5.61 in the matched comparisons. In the seventh year after the index date, the rates were 12.57 in fathers of children with cancer, 9.59 in fathers of matched comparisons, 18.66 in mothers of children with cancer, and 13.27 in mothers of matched comparisons.

### Mental health disorders for parents of children with cancer

By fitting the model, we aimed to examine the pattern of mental health outcomes for both mothers and fathers of children with cancer. From the parameter estimates shown in Table 2, we could see that the occurrence of hospital contacts for mental health disorders in parents increased significantly during the study period, regardless of the groups.

**Table 2:** Parameter estimates in models for parents of children with cancer.

a	Mothers				Fathers			
Parameters	Coef.	SE	95% CI	P value	Coef.	SE	95% CI	P value
$\beta_{1}$	–	–	–	–	−0.34	0.17	[−0.67,−0.01]	0.04
$\beta_{2}$	0.08	0.01	[0.07,0.10]	<.0001	0.10	0.01	[0.08,0.12]	<.0001
$\beta_{3}$	–	–	–	–	0.27	0.13	[0.01,0.53]	0.04
$\beta_{4}$	0.03	0.01	[0.01,0.05]	0.01	–	–	–	–

The models were adjusted for country of birth, education level, place of living, parents' ages, child's sex and CCI scores. $\beta_{1}$ indicates the baseline difference between two groups; $\beta_{2}$ indicates the population-based time-varying effect of mental health disorder diagnoses; $\beta_{3}$ indicates the effect of a short-term shock (an abrupt change) to case parents in levels of hospital contacts for mental health disorder in the index year; $\beta_{4}$ indicates the effect of a long-term shock (a gradual slope change) to case parents in mental health disorder hospital contacts.

**Fig. 2:**
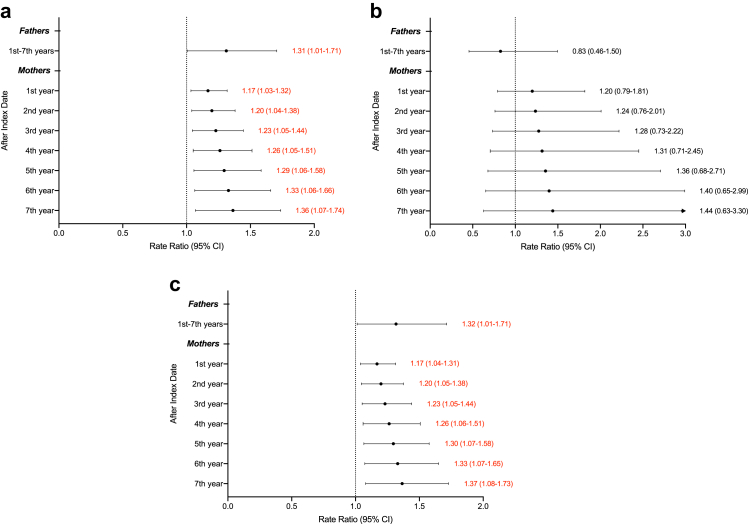
**The rate ratio of hospital contacts for mental health disorders in parents of children with cancer.** (a), all hospital contacts; (b), inpatient hospitalization; (c), outpatient visits. The analysis was adjusted for country of birth (Sweden, abroad), education level (≤9, 10–12, >12), place of living (big cities, other northern cities, other southern cities), parents' ages (continuous), child's sex (boy, girl) and CCI scores (continuous). The lines and values highlighted in red indicate statistical significance. The data within brackets indicate 95% confidence intervals (CIs) of the rate ratio.

**Fig. 3:**
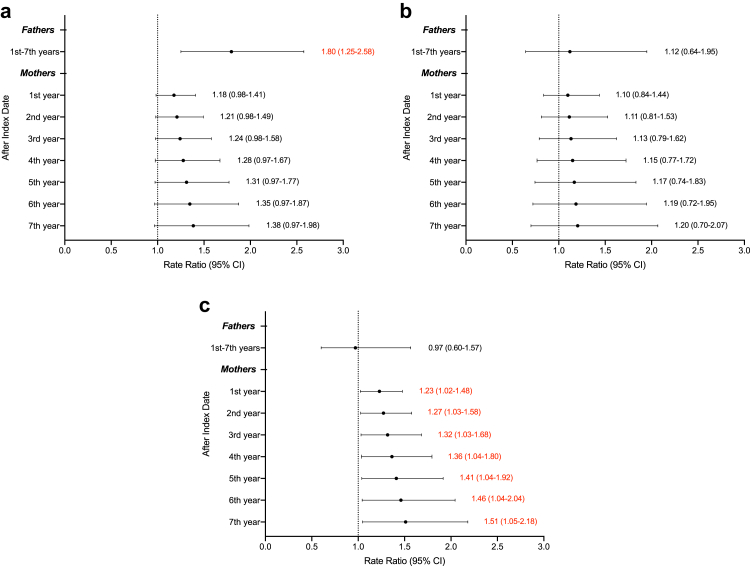
**The rate ratio of hospital contacts for mental health disorders in parents of children with cancer, stratified by age at diagnosis of childhood cancer.** (a), 0–4 years at diagnosis; (b), 5–9 years at diagnosis; (c), 10–14 years at diagnosis. The analysis was adjusted for country of birth (Sweden, abroad), education level (≤9, 10–12, >12), place of living (big cities, other northern cities, other southern cities), parents' ages (continuous), child's sex (boy, girl) and CCI scores (continuous). The lines and values highlighted in red indicate statistical significance. The data within brackets indicate 95% confidence intervals (CIs) of the rate ratio.

The fathers of children with cancer (Fig. 2), compared to the matched fathers, had 32% higher rates of hospital contact (RR, 1.31; 95% CI, 1.01-1.71). Paternal mental health underwent an adverse change in the year when their child was diagnosed with cancer, considering that the estimate of $\beta_{3}$ (Table 2) was 0.27, and it was statistically significant (*P* value = 0.04). However, the adverse effect was noted only for outpatient visits (Fig. 2). Additionally, the change in mental health was significant only for fathers whose child was diagnosed with cancer before age five (Fig. 3) and had cancer in other sites and not haematological malignancies or nervous system tumours (Fig. S1). For specific mental health disorders (Fig. S2), only severe depressive disorders (RR, 3.32; 95% CI, 1.06–10.39) and adjustment disorders (RR 2.99; 95% CI, 1.58–5.65) had significant associations.

However, the pattern of change in mental health outcomes for mothers was different from that of fathers (Fig. 2). Mothers of children with cancer were at higher risk compared to mothers in the reference group. Mothers’ mental health got worse in the long-term model, as determined by the statistical significance of the estimate of $\beta_{4}$ of 0.03 (*P* value = 0.01), shown in Table 2. The rate ratio was 1.17 (95% CI, 1.03–1.32) during the first year after diagnosis and, notably, increased by approximately 3% per year, reaching 1.36 (95% CI, 1.07–1.74) in the seventh year of follow-up. The adverse effect was, however, noted only for outpatient visits (Fig. 2). Furthermore, the association was significant only for the mothers of the child whose cancer was diagnosed between 10 and 14 years of age (Fig. 3). Almost all specific mental health disorders of interest showed a significant association (Fig. S3). Mothers of cancer-affected children were less likely to suffer from alcohol abuse (RR 0.56; 95% CI, 0.34–0.91 in the first year, and 0.31; 95% CI, 0.12–0.84 in the seventh year after diagnosis), but were at higher risks of having severe depressive disorders (first year: RR 2.15, 95% CI, 1.37–3.38; seventh year: RR 4.64, 95% CI, 1.88–11.41), recurrent depressive disorders (first year: RR 1.48, 95% CI, 1.18–1.85; seventh year: RR 2.18, 95% CI, 1.38–3.44), and adjustment disorders (first year: RR 1.56, 95% CI, 1.27–1.90; seventh year: RR 2.42, 95% CI, 1.62–3.62).

## Discussion

In this population-based cohort study, we explored the short- and long-term patterns of hospital contacts for mental health disorders among parents of children with cancer. Considering the differences in parental mental health before the cancer diagnosis in children, our study found that compared to matched parents, both mothers and fathers of children with cancer experienced a higher rate of mental health disorders. However, the patterns were different. The adverse effect on fathers was relatively stable and constant during the follow-up period, whereas the adverse effect became continuously worse for mothers. The main adverse effect for the parents was severe depressive disorders and adjustment disorders. Our study suggested that early and ongoing assessments of mental health are highly needed in parents after their children are diagnosed with cancer. Additionally, appropriate interventions, such as supportive social networks and the use of psycho-social services, could benefit and optimise parental well-being and family functioning.^16^^,^^17^

Previous studies found a higher risk of worse mental health among parents of children with cancer compared with the general population,^7^^,^^14^^,^^30^^,^^31^ which was also observed in our study. We found that fathers were affected immediately when their child was diagnosed with cancer. The risk of mental health disorders increased by 31% for fathers and remained relatively stable during the entire study period. A study using the insurance claims data of US families suggested a 66% increase in the risk of mental health diagnoses in fathers with two years of follow-up.^30^ In a Danish registry-based study, however, elevated risk of hospital contacts for psychiatric disorders was not observed.^18^ The differences in findings may be explained by the different sampling strategies and potential underestimation of mental health disorders. The samples of US families consisted entirely of those who had private insurance coverage, which might have the potential for overuse of health care resources.^32^ Additionally, mild to moderate mental health disorders may not be captured in the Danish Psychiatric Central Research Register.^18^

It was found that mothers encountered continuously elevated and frequent hospital contacts for mental health disorders. The rates of hospital contact for mental health disorders were 17% higher in the first year after a child was diagnosed with cancer, and the risk was increased to 36% six years later. A vicious cycle of high care burden,^33^ gradual physically and emotionally exhaustion, and exacerbated health problems in children may explain the ever-increasing risk of maternal mental health outcomes. No previous studies investigated the trends in risk levels of mental health-related hospital contacts, but we could gain similar insight into consequent mental health outcomes from the available literature. The US study found a 91% increase in the risk of mental health disorders.^30^ The two registry-based studies found a 23% increased risk of inferior mental health outcomes in Denmark and a 40% increase in rates of outpatient visits in Canada. Despite the varying degree of risk experienced by mothers, a tendency was found toward worse mental health outcomes compared with fathers. The different trajectories of mental health outcomes we observed in mothers and fathers could be explained as the result of a range of factors. It has been proved that sex difference characterises the incidence and vulnerability of numerous psychiatric disorders.^34^ However, we suggested that a different parenting role in the family might be a more appropriate explanation rather than biological mechanisms. In caring for a cancer-affected child, parental responsibilities were often unevenly distributed.^35^ Even in Sweden, with relative equality of duties between the parents, traditional parenting roles still predominated.^36^, ^37^, ^38^ Mothers usually took more caring tasks related to household work and children's medical care,^39^ requiring long-term commitments, whereas fathers are more expected to contribute financially.

Although we didn't observe significant differences in inpatient hospitalisation between parents of children with cancer and parents of cancer-free children, the outpatient visits differed more between groups. In addition, we were able to identify the most vulnerable subpopulations. Fathers of children diagnosed with cancer at ages 0–4 and mothers of children diagnosed with cancer at ages 10–14 were at a particularly high risk of developing mental health disorders. Most previous studies suggested that the parents of children diagnosed at a younger age were relatively more vulnerable, potentially attributed to a higher caregiving burden,^18^ which could explain our finding in fathers. Nevertheless, the specific role of mothers in the family might contribute to the relatively higher risk of mental health disorders among mothers of children who were diagnosed with cancer at the age of 10–14. A previous study showed that the caregiving burden was suggested positively associated with children's age, especially for the ill child with long-term care.^40^ Mothers who have more childcare tasks in the family are more vulnerable to a sudden aggravated care burden at the expense of normal routines. The increased worries about the late effects of cancer and fertility issues^41^ might also increase the risk of mental health problems in mothers whose children were diagnosed at an older age. Despite the knowledge that certain subtypes of childhood cancer have worse survival rates,^42^ childhood cancer sites were generally not differentially associated with the risk of maternal or paternal mental health outcomes. No association between childhood cancer sites and parental mental health outcomes was also observed in a previous study.^19^

Several specific mental health disorders were examined in our study. Risks were greatly elevated for severe depressive disorder and adjustment disorder among both mothers and fathers of children with cancer compared to reference parents; this was also observed in a prior study.^18^ Furthermore, among mothers of cancer-affected children, the risk of the recurrent depressive disorder increased significantly over time. We expected that parents of children with cancer might have the same pattern, identified in some chronic patient caregivers in previous studies, of being at risk for problematic alcohol use as they all have a heavy care burden and may experience social isolation.^43^^,^^44^ Interestingly, we found that mothers of children with cancer in Sweden would have a significantly decreased risk of alcohol abuse, whilst fathers had a slight reduction in the risk of alcohol abuse compared to the general population. One potential explanation is that the screening tools were used to define alcohol dependence in prior studies, instead of clinical diagnoses in our study. Social isolation due to their responsibility of caring for ill children lowers their social activity as well as the chance of alcohol intake.

The main strength of our study is the utilisation of Swedish nationwide administrative registers, which allowed us to conduct the analysis with high-quality data. In most previous studies, measures mainly examined short-term mental health conditions and relied upon self-reported symptoms or questionnaires to participants in small studies, which might lead to social desirability bias.^45^ The advantages of registers overcame these limitations and allowed us to obtain clinical measurements with long-term follow-up and adequate sample size, ensuring statistical power in the stratification analyses. The link to the registered data also enabled the selected reference group to be representative of the general population without selection bias. In addition, repeated measurements of hospital contacts allowed us to explore the temporal trends in the risk of parental mental health outcomes. In our analysis, no difference was found for the mothers on the slope of mental health-related hospital contact rates before the cancer diagnosis of the child. Surprisingly, we observed a slightly low rate of hospital contact among the fathers of children with cancer before the index date. It is known that the diagnosis of childhood cancer might take a long time from the initial symptom to the final diagnosis. Fathers could focus on their child's health status instead of going to hospitals for their self-health problems. This is also the reason why we prefer to use the interrupted time series analyses to account for the difference prior to the index date. Additionally, the ITS analytic frame that we used is good enough to remove the original difference in after-event period comparisons between two groups, regarding it as a result likely to represent a permanent difference.^28^ Moreover, the long observation period in our study enabled consistent estimations of time trends and parental comparisons.

However, our study also has several limitations. First, we may overestimate the clinical diagnoses of mental health disorders in parents. Hospital contacts for any mental health disorder might not be a perfect indicator of the need for psychological support, as repeated outpatient visits may represent prolonged self-willed intervention as opposed to sustained mental health disorders. The usage of medications to treat mental health conditions could be a better proxy to explore the prevalence of clinical mental health disorders, which should be further studied. However, we could still judge that the result of our study made it worthwhile to provide more psychological support for parents of children with cancer. Alternatively, we may also underestimate the burden of mental health problems because we only included hospital contacts. Even though data for hospital contacts covers both public and private healthcare systems in registers, we were unable to capture those who may have suffered from worse mental health without seeking any psychological support or who may have accessed support from services undetected by us, such as social support groups. In addition, some treatment-related variables, such as treatment duration and treatment regimens, as well as the information on whether the child died from cancer, were not considered in our study. Further studies could take these factors into account.

In conclusion, parental mental health was greatly and negatively affected by the child being diagnosed with cancer in Sweden. Given the lack of psychological support in a large proportion of parents of childhood cancer survivors, support from healthcare professionals should be considered for the parents during and years after the treatment, especially for those with a higher susceptibility, such as mothers whose child was diagnosed with cancer at an older age. Risks in mothers have been shown to increase continuously, calling for extra attention to the mothers such as supportive social networks and customised psychological services, to safeguard their mental well-being. Timely interventions could slow the progression of mental health problems and might finally lead to an improved prognosis for the child with cancer.

## Contributors

All authors were responsible for the study concept and design. JS, KS, and JJ obtained funding. KS and JS acquired the data. YL, DZ, and JJ did the statistical analysis, contributed to the interpretation of findings, and verified the underlying data. YL drafted the manuscript and manifested the visualization. And all authors read, discussed, critically revised the manuscript for intellectual content and approved the final version of the manuscript. JJ attested that all authors had full access to all data in the study, and had the final responsibility to submit it for publication.

## Data sharing statement

Data are available from the corresponding author upon reasonable request.

## Declaration of interests

The authors disclose no conflicts.
