# Supplementary material for: Mental health outcomes in parents of children with a cancer diagnosis in Sweden: A nationwide cohort study
Source: eClinicalMedicine. 2022 Nov 17;55:101734. doi: 10.1016/j.eclinm.2022.101734 (PMC9676277; doi:10.1016/j.eclinm.2022.101734)
Supplement: Multimedia component 1 [file mmc1.pdf]

Supplementary material

Mental health outcomes in parents of children with a cancer diagnosis in Sweden: a nationwide cohort study

Yishan Liu, Jan Sundquist, Kristina Sundquist, Deqiang Zheng, and Jianguang Ji

Contents	Pages
Figure S1: The rate ratio of hospital contacts for mental health disorders in parents of children with cancer, stratified by childhood cancer sites.	2
Figure S2: The rate ratio of specific types of mental health disorders in fathers of children with cancer	3
Figure S3: The rate ratio of specific types of mental health disorders in mothers of children with cancer	4

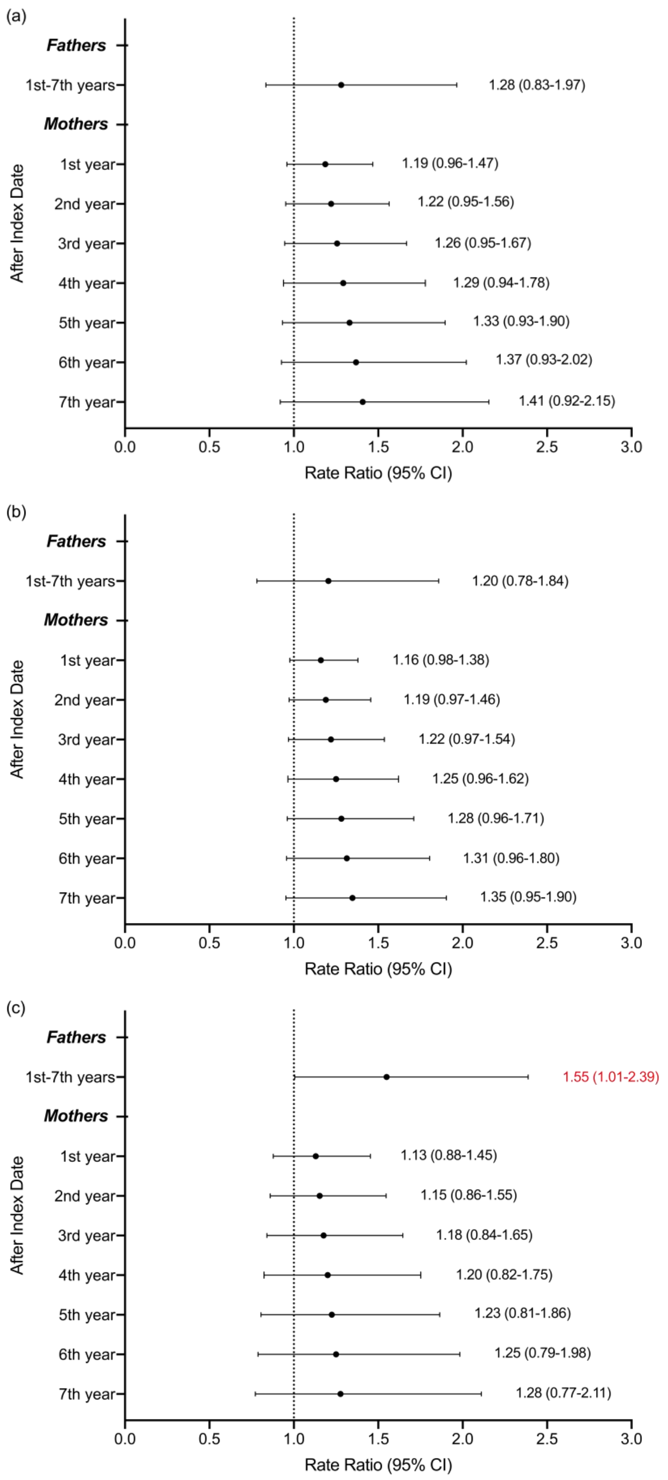

Figure S1. The rate ratio of hospital contacts for mental health disorders in parents of children with cancer, stratified by childhood cancer sites. (a), nervous system tumors; (b), hematological malignancies; (c), others. The analysis was adjusted for country of birth (Sweden, abroad), education level (<= 9, 10-12, >12), place of living (big cities, other northern cities, other southern cities), parents' ages (continuous), child's sex (boy, girl) and CCI scores (continuous). The lines and values highlighted in red indicate statistical significance. The data within brackets indicate 95% confidence intervals (CIs) of the rate ratio.

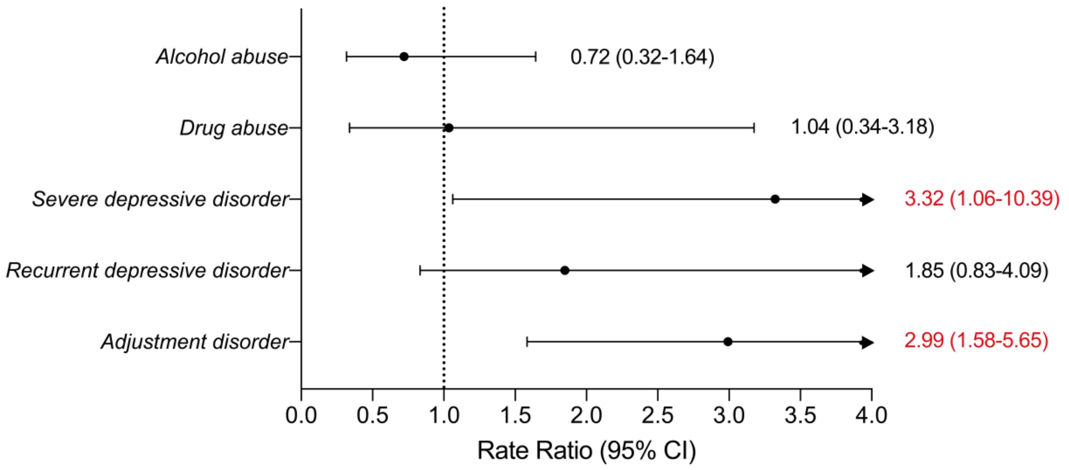

Figure S2. The rate ratio of specific types of mental health disorders in fathers of children with cancer. The analysis was adjusted for country of birth (Sweden, abroad), education level (<= 9, 10-12, >12), place of living (big cities, other northern cities, other southern cities), fathers' ages (continuous), child's sex (boy, girl) and CCI scores (continuous). The lines and values highlighted in red indicate statistical significance. The data within brackets indicate 95% confidence intervals (CIs) of the rate ratio.

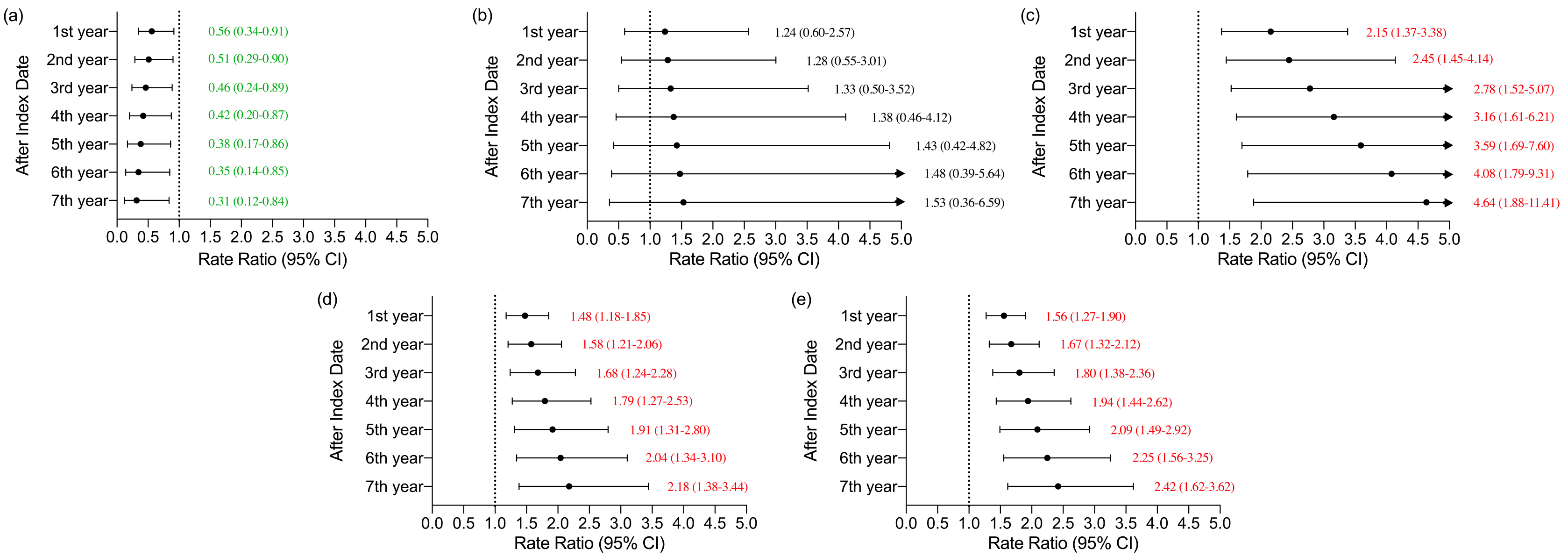

Figure S3. The rate ratio of specific types of mental health disorders in mothers of children with cancer. (a), alcohol abuse; (b), drug abuse; (c), severe depressive disorder; (d), recurrent depressive disorder; (e), adjustment disorder. The analysis was adjusted for country of birth (Sweden, abroad), education level (<= 9, 10-12, >12), place of living (big cities, other northern cities, other southern cities), mothers' ages (continuous), child's sex (boy, girl) and CCI scores (continuous). The lines and values highlighted in green indicate a lower risk compared to comparison parents with statistical significance. The lines and values highlighted in red indicate a higher risk compared to comparison parents with statistical significance. The data within brackets indicate 95% confidence intervals (CIs) of the rate ratio.
